# Cell-wall structural changes in wheat straw pretreated for bioethanol production

**DOI:** 10.1186/1754-6834-1-5

**Published:** 2008-04-16

**Authors:** Jan B Kristensen, Lisbeth G Thygesen, Claus Felby, Henning Jørgensen, Thomas Elder

**Affiliations:** 1 Forest and Landscape Denmark, University of Copenhagen, Rolighedsvej 23, DK-1958 Frederiksberg, Denmark; 2 Utilization of Southern Forest Products, USDA-Forest Service, Southern Research Station, Shreveport Highway, Pineville, LA 71360, USA

## Abstract

**Background:**

Pretreatment is an essential step in the enzymatic hydrolysis of biomass and subsequent production of bioethanol. Recent results indicate that only a mild pretreatment is necessary in an industrial, economically feasible system. The Integrated Biomass Utilisation System hydrothermal pretreatment process has previously been shown to be effective in preparing wheat straw for these processes without the application of additional chemicals. In the current work, the effect of the pretreatment on the straw cell-wall matrix and its components are characterised microscopically (atomic force microscopy and scanning electron microscopy) and spectroscopically (attenuated total reflectance Fourier transform infrared spectroscopy) in order to understand this increase in digestibility.

**Results:**

The hydrothermal pretreatment does not degrade the fibrillar structure of cellulose but causes profound lignin re-localisation. Results from the current work indicate that wax has been removed and hemicellulose has been partially removed. Similar changes were found in wheat straw pretreated by steam explosion.

**Conclusion:**

Results indicate that hydrothermal pretreatment increases the digestibility by increasing the accessibility of the cellulose through a re-localisation of lignin and a partial removal of hemicellulose, rather than by disruption of the cell wall.

## Background

Research in bioethanol production from lignocellulosic plant materials has grown significantly over the last few decades as the depletion of non-renewable fuels and increasing greenhouse gas emissions continue to create an increasing need for an alternative non-fossil transportation fuel. Enzymatic hydrolysis of lignocellulosic biomass, such as agricultural residues, with subsequent fermentation of sugars into ethanol has long been recognised as an alternative to the existing starch and sucrose-based ethanol production, especially considering recent improvements in yields and enzyme prices [1-3]. Furthermore, lignocellulose may be used as a feedstock for biorefineries, and full-scale plants for cellulosic bioethanol production are planned or under construction in several countries.

Two process steps are involved in the conversion of lignocellulose into bioethanol: (1) enzymatic hydrolysis of the cell-wall carbohydrates, cellulose and in some cases hemicellulose, into monomers; and (2) fermentation of the monomers into ethanol. Often the two processes are integrated into simultaneous saccharification and fermentation (SSF). A common feature of the enzymatic hydrolysis step is the need for pretreatment of the lignocellulosic material resulting in a more efficient reaction despite the recalcitrant nature of the plant cell wall [4].

While a costly step in production, optimal pretreatment is important from an economic viewpoint, as it has an impact on product yields and concentration, the rate of hydrolysis and fermentation, enzyme loading, waste products and fermentation toxicity [5]. The effect of the pretreatment has been described as a disruption of the cell-wall matrix including the connection between carbohydrates and lignin, as well as depolymerising and solubilising hemicellulose polymers [6]. This improves access for the saccharifying enzymes and alleviates mass-transport limitations [5]. Pretreatment is also able to change the degree of cellulose crystallinity [7].

There are several different ways of pretreating biomass, depending on the type, composition and subsequent processing technology that will be applied. The most widely investigated pretreatment technologies are thermochemical treatments such as dilute acid treatment (with or without rapid steam decompression (explosion)) [8-10] and ammonia pretreatment [11,12]. Hydrothermal pretreatment without the use of chemicals has also proven to be effective [13,14]. For a review of the most important pretreatment methods, see [5,15].

Recently, an EU-funded project on the co-production of bioethanol and electricity (Integrated Biomass Utilization System - IBUS) has resulted in a hydrothermal pretreatment process for wheat straw that has proven to be effective at preparing straw for enzymatic hydrolysis [16]. The process is designed to handle large particles (pieces of straw over 5 cm in length) and run at high dry-matter levels (exceeding 30% w/w) [16]. In the process, the straw is treated with water while being moved through a counter-current reactor at a temperature of 190-200°C. The wash water can be recycled and salt and solubilised hemicellulose sugars can be isolated [16]. A pretreatment pilot plant with a capacity of up to 1000 kg/hour has been working since 2006. As described in [16] and [17], the pretreated straw can be enzymatically liquefied, saccharified and subsequently fermented into ethanol at initial dry-matter levels of up to 40% w/w. Recent SSF experiments with an initial dry-matter content of 27% (w/w) have produced ethanol levels of over 60 g/kg slurry [18]

Atomic force microscopy (AFM) has proven to be a powerful tool for visualising the surface of plant cell walls [19-22] including modification of plant fibres and pulp [23-25]. In the present study, AFM and scanning electron microscopy (SEM) investigations of the effects of hydrothermal treatment on straw cell wall disruption, composition, ultrastructure and surface properties were carried out in order to better understand the increased susceptibility to enzymatic hydrolysis. Chemical decomposition into constituent polymer classes was carried out for all sample types. Attenuated total reflectance Fourier transform infrared (ATR-FTIR) spectroscopy was used as an analytical tool to qualitatively determine the chemical changes in the lignocellulosic material upon pretreatment. For comparison, analyses were also carried out on SO_2_-impregnated steam-explosion pretreated wheat straw. Steam explosion is a widely recognised pretreatment [8].

## Results and discussion

### Straw composition

As seen in Table 1, the main effect of the hydrothermal pretreatment on the composition of the biomass is the partial but substantial removal of hemicelluloses. All measurable arabinan is removed and the xylan content is reduced from 24.5% to 5.2%. Consequently, the overall cellulose content increases. After delignification of the pretreated material, no Klason lignin can be detected. The composition of the straw that has undergone SO_2_-impregnated steam explosion is similar to that of the hydrothermally pretreated straw except for a slightly higher xylan content at 7.8%.

**Table 1:** Compositions

	Cellulose	Xylan	Arabinan	Klason lignin	Ash
Straw, untreated	39.8	24.5	2.8	22.6	4.2
Pretreated straw	59.0	5.2	0.0	25.5	5.6
Delignified, pretreated straw	75.1	9.8	0.0	0.0	8.8
Steam-exploded straw	56.7	7.8	0.7	23.6	6.3

Contents expressed as percentages, based on dry matter.

### ATR-FTIR spectroscopic analysis

**Figure 1:**
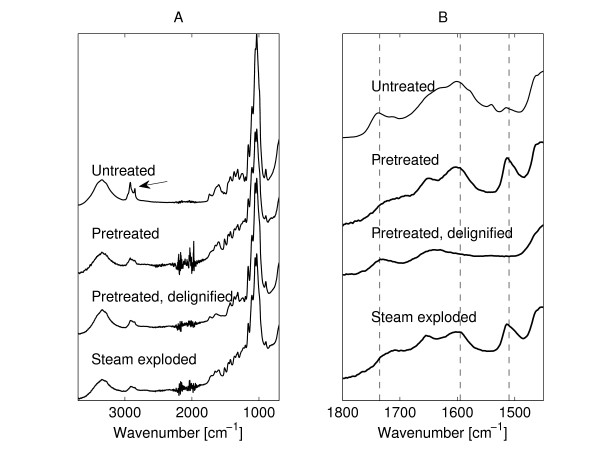
**Spectroscopy**. ATR-FTIR spectra of untreated, hydrothermally pretreated, delignified hydrothermally pretreated and steam-exploded wheat straw. **(A) **Complete spectra of all treatments. **(B) **Excerpt of spectra. All spectra are separated to ease comparison. The arrow in A points to the bands at 2850 and 2920 cm^-1 ^(CH_2_- stretching bands ascribed to wax). The vertical lines in B mark the positions of the bands at 1735 (carbonyl, ascribed to hemicellulose), 1595 and 1510 cm^-1 ^(aromatic ring stretch, ascribed to lignin).

ATR-FTIR spectroscopy was used as an analytical tool to qualitatively determine the chemical changes in the surface of pretreated straw to complement and understand the microscopic investigations. The FTIR spectra of untreated, hydrothermally pretreated, delignified hydrothermally pretreated and steam-exploded straw samples are shown in Fig. 1A. Excerpts of the four spectra are presented in Fig. 1B.

One of the effects of the pretreatment is the removal of wax from the straw: Fig. 1A shows that the CH_2_- stretching bands at approximately 2850 and 2920 cm^-1 ^(see [26]) are reduced for the pretreated straw sample, signifying a reduction in the amount of the aliphatic fractions of waxes.

Two interesting features are shown in Fig. 1B. First, it can be seen that the carbonyl band at 1735 cm^-1^, which has been ascribed to hemicelluloses [27-29] is reduced for the pretreated straw. This is expected as the pretreatment is known to remove a large portion of the hemicelluloses as shown in Table 1 and in Thomsen et al. [16]. Second, lignin bands at approximately 1595 and, in particular, 1510 cm^-1 ^(aromatic ring stretch) [30] are strongly enhanced in the hydrothermally pretreated sample compared with both untreated wheat straw and delignified hydrothermally pretreated straw, where these peaks are reduced (Fig. 1B). One explanation for this could be a relative increase in the amount of lignin due to the removal of hemicelluloses. Another reason could be that lignin is released and re-deposited on the surface (ATR-FTIR spectroscopy is a surface technique; according to [26,31] the penetration depth in straw is approximately 0.5-3 μm with the signal intensity exponentially decreasing with penetration depth). The increase in lignin is believed to be too significant to be only due to the hemicellulose removal.

One of the strategies employed in increasing enzymatic convertibility is to decrease cellulose crystallinity [15]. Differences between samples with regard to the relative amounts of amorphous and crystalline cellulose have earlier been described through infrared peak ratios. At least four different peak pairs have been proposed [32,33]. Of these, only the peak pair 1429 cm^-1 ^(crystalline) and 893 cm^-1 ^(amorphous) is seen for the samples of the present study. The peak ratio for the untreated straw was 0.56, while it was 0.52 for the pretreated straw. In the study by Wistara et al. [33], values from 0.46 to 0.56 were reported, and from this and other results the authors claimed that there was no difference in crystallinity between their samples. When comparing their results with ours, it appears that the pretreatment does not adversely affect the degree of cellulose crystallinity. More precise measurements of cellulose crystallinity are needed to confirm this result.

### SEM and AFM images

Based on the results from ATR-FTIR spectroscopy, SEM and AFM were used to gather information on the effect of the hydrothermal pretreatment on the ultrastructure and possible disruption of the cell wall.

**Figure 2:**
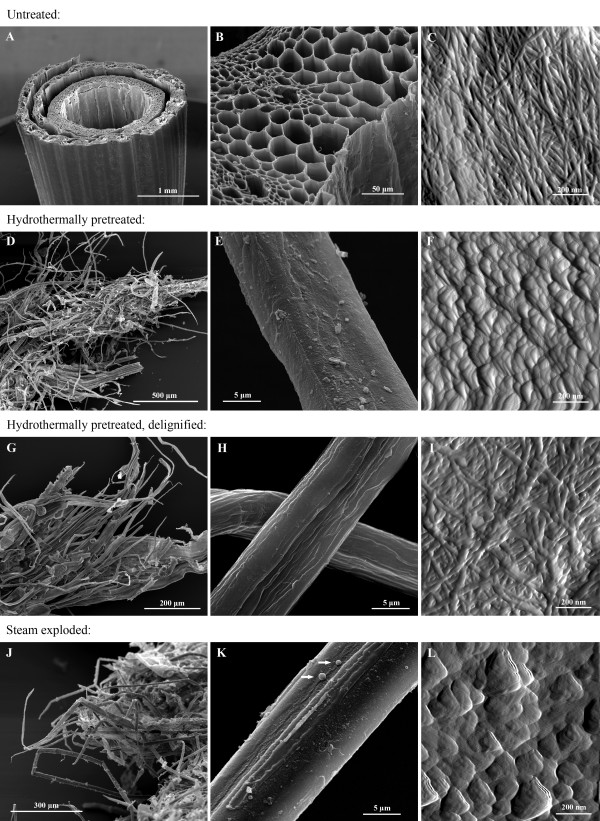
**Microscopy images**. SEM and AFM images of untreated **(A)**-**(C)**, hydrothermally pretreated **(D)**-**(F)**, delignified hydrothermally pretreated **(G)**-**(I) **and steam-exploded wheat straw **(J)**-**(L)**. In untreated wheat straw, the straw itself is surrounded by a sheath leaf (A, SEM image) and at slightly higher magnification the individual cells of the straw wall can be identified (B, SEM image). A high-resolution AFM scan (amplitude image) of a primary cell wall lining the straw cavity shows interwoven cellulose microfibrils, partially imbedded in non-cellulosic polymers (left-hand side of C). In hydrothermally pretreated wheat straw, the defibrating effect of the pretreatment causes the individual fibres to partially separate, as can be seen in D (SEM image). The pretreatment leaves a surface layer of debris and re-deposited cell-wall polymers on the individual fibres (E, SEM image). An AFM scan (amplitude image) of fibre surface shows the 'globular' deposits characteristic of lignin (F). No microfibrils are visible. Delignification of pretreated fibres causes no further separation of fibres (G and H, SEM images) but removes most of the surface layer/deposits seen in (E). Cellulose lamellae/agglomerates are now visible (H). An AFM scan (amplitude image) shows that delignification exposes intact, interwoven cellulose microfibrils (I). Steam explosion causes partially separated fibres with 90° compression bends (J, SEM image) and a surface layer with debris and droplets (K, SEM image). Droplets are indicated with arrows. High-resolution imaging of AFM shows globular surface deposits (L, amplitude image), similar to those seen on hydrothermally pretreated straw (F).

When untreated, the anatomy of the harvested, chopped wheat straw is easily recognisable, with sheath leaves surrounding the straw itself (Fig. 2A). The various cell types of the straw wall can be seen, including epidermis cells, parenchyma cells, vascular bundles (phloem and xylem) as well as thick-walled fibre cells, as seen in the SEM micrograph presented in Fig. 2B. Imaging by AFM of parenchyma cells lining the straw cavity reveals the appearance of interwoven cellulose microfibrils of the primary wall (Fig. 2C). These particular cells are largely unlignified [34] but microfibrils are partially embedded in what is believed to be hemicellulosic polymers (left-hand side of Fig. 2C).

Initially, the most apparent effect of the hydrothermal pretreatment apart from a colour change from yellow into dark brown is the partial defibration, or separation of individual fibres and cell types of the wheat straw. Although the pretreated material is quite heterogeneous and contains larger pieces (up to about 1 cm) that are easily recognised as straw, a significant fraction consists of cells that are either completely or partially separated from each other (Fig. 2D).

All individual fibres (and most other cell types) seem to be intact despite the hydrothermal treatment, rather than being broken or otherwise disrupted (Fig. 2D and 2E). When looking more closely at the pretreated fibres it becomes apparent that the surface is covered with 'debris' and a thin layer of deposits that seems to be covering the whole surface (Fig. 2E). This debris could be fractions of middle lamellae. When further investigating the pretreated fibre surfaces through AFM, it was not possible to identify any primary or secondary wall cellulose microfibrils (such as seen in untreated fibre cell walls; Fig. 2C). Instead, an uneven surface of spherical and globular shapes was seen (Fig. 2F). These globular shapes (diameter approximately 20-100 nm) are characteristic of lignin deposits as reported in the literature [22,25,35], and this interpretation is in accordance with the spectroscopic findings of higher surface lignin concentrations.

Initially, delignification did not have a great effect on the overall structure of the pretreated material apart from a change in colour; the straw was still only partially defibrated (Fig. 2G), presumably due to the hemicellulose content of the middle lamella [34]. However, upon closer observation, the surface of the individual fibres had changed drastically. The uneven surface now appeared smooth and cellulose aggregates (macrofibrils) running in the direction of the fibre could be seen, as in the SEM image in Fig. 2H. When investigating the delignified fibre surfaces with AFM, the globular shapes of deposited lignin were not seen. Instead, intact surfaces believed to be primary and secondary wall lamellae were observed. Due to the mixing of fibres and other cell types during the pretreatment it was not possible to investigate the same straw cavity parenchyma cells as with the untreated straw. However, numerous scans of different cells revealed several surfaces with similar primary walls to the parenchyma cells. The microfibrils of these primary walls displayed the same interwoven structure as previously seen and were partially embedded in non-cellulosic polymers (Fig. 2I). It should be added, that with AFM only relatively smooth surfaces are successfully imaged.

Surprisingly, neither the overall or fibrillar structure of the individual fibres seems to show large structural changes such as the rupture of fibres or a visible increase of porosity, which are believed to be associated with thermal pretreatments. No holes or cracks were seen in the fibres and AFM did not indicate that the accessibility of the internal parts of the cell wall matrix had been improved due to structural dislocations. Rather, the primary and secondary cell walls appeared to be fully intact, except for the pits and simple perforations that already exist in certain cell types [36]. Despite these observations of a substrate where the skeletal structure is intact and the crystallinity of the cellulose does not appear to have been lowered, the hydrothermally pretreated straw has been shown to be easily digestible by enzymes [16,17]. Consequently, the effectiveness of the pretreatment must be related to hemicellulose removal and lignin re-localisation. This is in spite of the fact that lignin is not removed by the pretreatment and that lignin is known to be responsible for unproductive adsorption of cellulases [37,38]. It is well known that lignin encases the cellulose in the cell-wall matrix, hindering cellulases from reaching cellulose fibrils. We hypothesise that the migration of lignin to the outer surface exposes internal cellulose surfaces. More investigations are needed in order to confirm this. Selig et al. [39] have also observed the formation and migration of spherical lignin deposits onto the surface of fibres as a result of pretreatment. They also suggest that the deposited lignin can have a negative impact on the enzymatic cellulose hydrolysis. It is possible, however, that the surface lignin layer is easily removed by simple mechanical forces through mixing, due to lignin being less strongly bound to carbohydrate polymers compared with its native linkages. Furthermore, we theorise that the re-located lignin has exposed cellulose inside the cell wall, thus increasing the enzyme accessibility.

Based on these observations, we therefore propose that the re-localisation of lignin as well as partial hemicellulose removal are likely to be important factors in increasing the enzymatic digestibility of wheat straw through hydrothermal pretreatment. It seems that exposing cellulose through manipulation of hemicelluloses and lignin are equally as important as altering the crystallinity and rupture of the skeletal structure of the cell wall.

### Comparison with conventional steam explosion

In order to understand whether the factors affecting biomass digestibility through hydrothermal pretreatment are of a more general nature, steam-exploded straw was also investigated microscopically and spectroscopically. Steam explosion is considered one of the most promising pretreatment technologies and is often combined with the addition of chemicals [5,6]. In our case the straw was impregnated with SO_2 _prior to steam explosion. In principle, steam explosion is not unlike hydrothermal pretreatment. As such, the effect on compositional changes is also similar (Table 1).

As seen in Fig. 1 the FTIR spectra of the steam-exploded straw are similar to those of hydrothermally treated straw, both in general and in spectral ranges related to wax, hemicellulose and lignin. SEM investigations (Fig. 2J and 2K) showed that steam-exploded straw was more heterogeneous than hydrothermally pretreated straw, containing larger pieces of almost intact straw but also a larger fraction of individual fibres that had been compacted together. Some SEM images also showed droplets on the surface of the fibres (see the arrows in Fig. 2K). These droplets are also believed to be lignin, possibly formed through coalescence of smaller sized lignin deposits during the pretreatment as described in [39]. The difference in amount of larger lignin droplets between the different pretreatments may be due to varying water contents and pH during the treatment. AFM showed globular deposits similar to, but larger than those seen on hydrothermally pretreated straw (Fig. 2L).

## Conclusion

Hydrothermal pretreatment has proven to be an effective way of increasing the enzymatic digestibility of wheat straw for conversion into fermentable sugars for bioethanol production. However, it has been unclear how the pretreatment affects the ultrastructure and molecular organisation of the biomass.

It was found that the hydrothermal pretreatment had a partial defibrating effect on wheat straw, producing a heterogeneous substrate of semi-separated fibres. Interestingly, in contrast to what might be expected, individual fibres were intact with no evidence of disruption. It was found that the vast majority of all fibre surfaces (more than 90%) were covered with a layer of globular deposits. The deposits were established to be re-localised lignin. Upon delignification of pretreated fibres, the cellulose fibrillar structure of the cell walls was found to be intact. The conservation of the skeletal structure of the cell wall through pretreatment is not in accordance with the general perception that pretreatments must disrupt the structure of the cell wall in order to increase its accessibility to enzymes.

Partial hemicellulose removal and lignin re-localisation are important factors in increasing the digestibility of hydrothermally pretreated wheat straw, possibly more important than rupture of the skeletal cell-wall structure and modification of cellulose crystallinity. Results show that it is possible to pretreat wheat straw sufficiently without disrupting the cell wall. Thus, only a modest pretreatment is necessary in order to enzymatically digest the carbohydrates, provided that mixing is efficient [17].

Although much is known about the chemical changes caused by pretreatments of lignocellulose, little seems to be known of the physical changes. We believe that research and development of technologies must be accompanied by structural and molecular investigations of the biomass in order to achieve substantial progress.

## Methods

### Pretreatments

The hydrothermal pretreatment was carried out at the IBUS pilot plant at Fynsværket in Odense, Denmark [16]. Pretreatment was performed at a feed rate of 75 kg of chopped wheat straw (0-5 cm long pieces) per hour (or approximately 67.5 kg dry matter per hour), which is pre-soaked in water at 80°C for 6 minutes prior to being transported into the reactor. Residence time in the reactor averaged 6 minutes with the reactor temperature maintained at 195°C by injection of steam, and a counter-current flow of water of 250 litres per hour. No chemicals were added to the water or the steam. The dry-matter content of the pretreated straw out of the reactor was between 25% and 32% (w/w). The pretreated straw was collected in plastic bags containing 30-50 kg of material and stored at 4°C for up to 5 months. The pretreated straw is used for hydrolysis and fermentation into bioethanol without any further treatment such as washing. For more information on this particular pretreatment technology as well as hydrolysis and fermentation studies with the pretreated material, see [16] and [17].

The steam-exploded straw was a gift from the Center for Chemistry and Chemical Engineering, Lund University, Sweden. The straw was pretreated as described in [38].

### Compositional analysis of straw

The typical compositions of straw, pretreated straw, delignified pretreated straw and conventionally steam-exploded straw were analysed using two-step acid hydrolysis according to the procedure published by NREL [40]. Before hydrolysis, the samples were dried at 45°C for 1 day. The dried samples were milled in a Braun coffee grinder. Dry matter was determined using a Sartorius MA 30 moisture analyser at 105°C. The content of monosaccharides in the hydrolysed samples (D-glucose, D-xylose and L-arabinose) was quantified on a Dionex Summit high-performance liquid chromatography (HPLC) system equipped with a Shimadzu RI-detector. The separation was performed in a Phenomenex Rezex RHM column at 80°C with 5 mM H_2_SO_4 _as an eluent at a flow rate of 0.6 ml min^-1^. Samples were filtered through a 0.45 μm filter and diluted with eluent before analysis on HPLC.

### Straw sample preparation for analyses

The straw samples analysed in this study consisted of an untreated control, hydrothermally pretreated material and steam-exploded straw, all of which were oven dried at 50°C for 24 hours. The hydrothermally pretreated straw was subsequently delignified by mixing approximately 25 g of dried straw with 800 ml MilliQ water, 40 ml of 98% glacial acetic acid and 20 g of sodium chlorite (NaClO_2_). The mixture was placed in a water bath at 80°C for 1 hour. The sodium chlorite and acetic acid additions were repeated twice, the second time with the addition of glacial acetic acid only. The reaction was terminated by cooling to 10°C. The holocellulose was isolated by filtration through a glass filter and rinsing with ice-cold MilliQ water, followed by oven-drying at 50°C for 24 hours. For SEM, the straw was lyophilised without prior oven-drying.

### ATR-FTIR spectroscopic analysis

ATR-FTIR spectra (4000-700 cm^-1^) were obtained using an ABB Bomem FTIR spectrometer equipped with a SensIR/Durascope diamond. An ATR accessory was used to qualitatively identify chemical changes in the pretreated wheat straw. Spectra were obtained with 4 cm^-1 ^resolution, and 128 scans for the background spectrum and 64 scans for each sample spectrum were performed. After drying, the straw sample was pressed against the diamond surface using a spring-loaded anvil to obtain the same pressure for each sample. To ensure that the surfaces measured were similar to those investigated by microscopy, the samples were not homogenised prior to spectral analysis. The risk taken when selecting this procedure was that the surface cells of the untreated straw were not representative for the bulk material. In order to check whether this was the case, some untreated material was ground to a fine powder, and ATR-FTIR spectra were obtained from the homogenised material. No significant differences were found between these spectra and those from the non-homogenised samples.

Spectra were recorded from three different sub-samples per sample type, and all spectra were corrected according to the standard normal variate (SNV) method [41]. The mean spectrum of the three corrected spectra is presented for each sample type.

### SEM analysis

SEM analysis was performed with a FEI Quanta 200 (FEI Company, Eindhoven, The Netherlands) operated at 20 kV. The samples were coated (gold/palladium) with a SC7640 Suto/Manual High Resolution Sputter Coater (Quorum Technologies, Newhaven, UK).

### AFM analysis

All AFM measurements were made with a MultiMode scanning probe microscope with a Nanoscope IIIa controller (Veeco Instruments Inc, Santa Barbara, CA). Images were acquired in TappingMode with an etched silicon probe (MPP-12100, Veeco NanoProbe, Santa Barbara, CA). An auto-tuning resonance frequency range of approximately 150-300 kHz with a scan rate of 0.5-3 Hz (usually around 2 Hz) was used. The drive amplitude and amplitude set-point were adjusted during measurements to minimise scanning artefacts. Height, amplitude and phase images were captured simultaneously. Scan size varied from 500 nm to 5.0 μm but was usually 1 μm.

Samples were fixed on metal discs with double-sided adhesive tape. All images were measured in air. Images were collected from a minimum of 20 different fibres for each treatment with representative images displayed in the present paper. To eliminate external vibration noise, the microscope was placed on an active vibration-damping table. All AFM images were recorded in a 512 × 512 pixel format and analysed and processed (contrast, illumination and plane fitting) by the accompanying Veeco Nanoscope software.

## Competing interests

The authors declare that they have no competing interests.

## Authors' contributions

JBK carried out the AFM and SEM work, participated in the spectroscopic measurements and drafted the manuscript. LGT carried out the spectroscopic measurements, analysed the spectra and drafted most of the spectroscopically related parts of the manuscript. CF participated in the AFM work as well as design and coordination of the study. HJ carried out the composition analyses, participated in the SEM work and helped to draft the manuscript. TE participated in the AFM work and the experimental design. All authors suggested modifications to the draft, commented on several preliminary versions of the text and approved the final manuscript
